# Tetanus in adult males, Bugando Medical Centre, United Republic of Tanzania

**DOI:** 10.2471/BLT.16.185546

**Published:** 2017-10-03

**Authors:** Riaz Aziz, Robert N Peck, Samuel Kalluvya, Bernard Kenemo, Alphonce Chandika, Jennifer A Downs

**Affiliations:** a Intensive Care Department, Bugando Medical Centre, PO Box 3750, Mwanza, United Republic of Tanzania.; b Department of Medicine, Weill Cornell Medicine, New York, United States of America.

## Abstract

**Problem:**

In the United Republic of Tanzania, the incidence of non-neonatal circumcision-related tetanus is probably underreported.

**Approach:**

We analysed charts and extracted information on outcome and wound location for non-neonatal cases of tetanus admitted to the intensive care unit of Bugando Medical Centre between 2001 and 2016.

**Local setting:**

Bugando Medical Centre, which is one of four teaching referral hospitals in the United Republic of Tanzania, has a 13-bed intensive care unit that manages all admitted patients with tetanus. Within the United Republic of Tanzania, formal programmes of tetanus immunization are targeted at infants or women.

**Relevant changes:**

From our inpatient logs, we identified six patients with non-neonatal tetanus among male patients with a recent history of circumcision. Only one of these patients had been circumcised within a subnational programme of voluntary medical male circumcision. The other five had been circumcised outside of the programme – e.g. at small rural dispensaries or by a traditional provider with no formal medical training. The six patients were aged 11–55 years and five (83%) of them died in hospital – all of overwhelming sepsis.

**Lessons learnt:**

Within the Tanzanian programme of voluntary medical male circumcision, education on wound hygiene probably helps to reduce the incidence of non-neonatal circumcision-related tetanus. The corresponding incidence among the boys and men who are circumcised beyond this subnational programme is probably higher. The training of all circumcision providers in wound care and a vaccination programme to ensure that male Tanzanians receive tetanus immunization post-infancy are recommended.

## Résumé

**Problème:**

En République-Unie de Tanzanie, l'incidence du tétanos non néonatal lié à la circoncision est probablement sous-estimée.

**Approche:**

Nous avons analysé des tableaux et extrait des informations concernant les suites et l'emplacement de la plaie des cas de tétanos non néonatal admis dans le service de soins intensifs du centre médical Bugando entre 2001 et 2016.

**Environnement local:**

Le centre médical Bugando, l'un des quatre hôpitaux centraux d'enseignement de la République-Unie de Tanzanie, dispose d'un service de soins intensifs doté de 13 lits qui prend en charge tous les patients admis avec le tétanos. Dans la République-Unie de Tanzanie, les programmes formels de vaccination contre le tétanos ciblent les nourrissons ou les femmes.

**Changements significatifs:**

Nous avons relevé, dans nos registres d'hospitalisation, six patients atteints de tétanos non néonatal parmi les patients masculins ayant récemment subi une circoncision. Seul un de ces patients avait été circoncis dans le cadre d'un programme infranational de circoncision masculine médicale volontaire. Les cinq autres avaient été circoncis en-dehors de ce programme, par exemple dans de petits dispensaires ruraux ou par un praticien traditionnel sans formation médicale formelle. Ces six patients étaient âgés de 11 à 55 ans et cinq (83%) d'entre eux sont décédés à l'hôpital, tous de septicémie.

**Leçons tirées:**

Dans le cadre du programme tanzanien de circoncision masculine médicale volontaire, l'éducation à l'hygiène de la plaie contribue probablement à réduire l'incidence du tétanos non néonatal lié à la circoncision. L'incidence correspondante chez les garçons et les hommes qui sont circoncis en-dehors de ce programme infranational est probablement plus importante. La formation en matière de soin des plaies de toutes les personnes qui pratiquent des circoncisions et la mise en place d'un programme de vaccination permettant aux Tanzaniens de bénéficier de vaccins contre le tétanos après la petite enfance sont recommandées.

## Resumen

**Problema:**

En la República Unida de Tanzania, es posible que no se comunique la incidencia del tétanos relacionado con la circuncisión no neonatal.

**Enfoque:**

Hemos analizado gráficos y extraído información sobre las consecuencias y la localización de la herida para los casos no neonatales de tétanos ingresados en la unidad de cuidados intensivos del Bugando Medical Centre entre 2001 y 2016.

**Marco regional:**

El Bugando Medical Centre, uno de los cuatro hospitales de referencia en la República Unida de Tanzania, cuenta con una unidad de cuidados intensivos de 13 camas que se ocupa de todos los pacientes ingresados con tétanos. En la República Unida de Tanzania, los programas oficiales de vacunación contra el tétanos están orientados a infantes o mujeres.

**Cambios relevantes:**

A partir de los registros de pacientes hospitalizados, se identificaron a seis pacientes con tétanos no neonatal entre los pacientes varones con un historial reciente de circuncisión. Solo uno de estos pacientes había sido circuncidado dentro de un programa subnacional para la circuncisión masculina voluntaria médica. Los otros cinco habían sido circuncidados fuera del programa, por ejemplo, en pequeños dispensarios rurales o atendidos por un proveedor tradicional sin formación médica oficial. Los seis pacientes tenían entre 11 y 55 años de edad y cinco (83%) de ellos murieron en el hospital, todos a causa de una sepsis incontenible.

**Lecciones aprendidas:**

Dentro del programa de Tanzania para la circuncisión masculina voluntaria médica, probablemente la educación sobre la higiene de las heridas ayude a reducir la incidencia del tétanos relacionado con la circuncisión no neonatal. Es probable que la incidencia correspondiente entre los niños y hombres circuncidados fuera de este programa subnacional sea mayor. Se recomienda la formación de todos los proveedores de circuncisión en el cuidado de heridas y un programa de vacunación para asegurar que los varones de Tanzania sean vacunados contra el tétanos después de la infancia.

## ملخص

**المشكلة:**

من المرجح وجود قصور في الإبلاغ عن حدوث الإصابة بالكزاز (التيتانوس) غير المرتبط بالولادة في جمهورية تنزانيا المتحدة.

**الأسلوب:**

قمنا بتحليل الرسوم البيانية واستخلاص المعلومات المتعلقة بنتائج ومواقع الجروح الخاصة بحالات الإصابة بالكزاز في غير حالات الأطفال حديثي الولادة الخاضعة للعناية المركزة في مركز بوغاندو الطبي بين عامي 2001 و2016.

**المواقع المحلية:**

يُعد مركز بوغاندو الطبي واحدًا من أربع مستشفيات جامعية تستقبل التحويلات الطبية في جمهورية تنزانيا المتحدة، كما أنه مجهز بوحدة للعناية المركزة تشتمل على 13 سريرًا للتعامل مع جميع حالات الإصابة بالكزاز التي يتم قبولها. وفي جمهورية تنزانيا المتحدة، تستهدف البرامج الرسمية لتمنيع الكزاز الرضع أو النساء.

**التغيّرات ذات الصلة:**

حددنا من بين سجلات المرضى الداخليين لدينا ستة حالات إصابة بالكزاز من غير الحالات المرتبطة بالأطفال حديثي الولادة وذلك بين المرضى الذكور الذين خضعوا مؤخرًا للختان. ولم يخضع سوى مريض فقط من بين هؤلاء المرضى للختان في إطار برنامج دون وطني لختان الذكور التطوعي. وقد تم ختان الخمسة الآخرين خارج نطاق البرنامج – على سبيل المثال في المستوصفات الريفية الصغيرة أو من قبل مقدم تقليدي للخدمات الطبية دون تدريب طبي رسمي. وتتراوح أعمار المرضى الستة ما بين 11 إلى 55 عامًا، وتوفي خمسة منهم (‏83‏%) في المستشفى بسبب الإنتان الشديد.

**الدروس المستفادة:**

من المرجح أن تفيد التوعية بشأن نظافة الجروح في الحد من الإصابة بالكزاز الناتج عن الختان وغير المرتبط بحديثي الولادة، وذلك في إطار البرنامج التنزاني للختان الطبي التطوعي للذكور. ومن المرجح أن تكون النسبة المقابلة بين الأولاد والرجال الذين يتم ختانهم خارج هذا نطاق البرنامج دون الوطني أعلى. ويوصى بتدريب جميع القائمين على إجراء الختان على كيفية العناية بالجروح وبرنامج التطعيم لضمان حصول الذكور التنزانيين على التمنيع ضد الكزاز بعد مرحلة الطفولة.

## 摘要

**问题:**

在坦桑尼亚联合共和国,与非新生儿割礼相关的破伤风发病率可能少报了。

**方法:**

我们分析了关于 2001 年至 2016 年间入住布坎多医疗中心 (Bugando Medical Centre) 重症监护病房的非新生儿破伤风病例的治疗效果和伤口位置的图表,并提取了信息。

**当地状况:**

作为坦桑尼亚联合共和国四家教学类转诊医院之一的布坎多医疗中心 (Bugando Medical Centre) 拥有 13 个床位的重症监护病房,管理所有入院的破伤风患者。坦桑尼亚联合共和国正式的破伤风免疫计划目标人群是婴儿或女性。

**相关变化:**

根据我们住院患者记录,我们在近期进行过割礼的男性患者中确定了六位非新生儿破伤风患者。其中只有一名患者是通过地方性自愿医疗男性割礼计划实施割礼的。其余五人均非通过该计划实施割礼 – 例如:通过小型的农村诊疗所,或通过没有经过正式医疗培训的传统提供者。这六位患者年龄在 11–55 岁之间,其中五人 (83%) 在医院死亡——全部为爆发性败血症。

**经验教训:**

坦桑尼亚联合共和国自愿医疗男性割礼计划以及伤口卫生教育可能有助于降低非新生儿与割礼相关的破伤风患病率。未通过这项地方性计划实施割礼的男孩和男性的相应患病率可能会增加。应面向所有割礼提供者进行关于伤口护理的培训,并推荐实施一项可确保坦桑尼亚男性居民在婴儿期之后的阶段接受破伤风免疫的免疫接种计划。

## Резюме

**Проблема:**

Показатели заболеваемости столбняком, связанным с обрезанием крайней плоти, проводимым не над младенцами, в Объединенной Республике Танзания, вероятно, являются заниженными.

**Подход:**

Мы проанализировали графики и получили данные об исходах и местоположении раны для случаев заболеваемости столбняком у пациентов старше младенческого возраста, поступивших в отделение интенсивной терапии Медицинского центра Бугандо в период с 2001 по 2016 год.

**Местные условия:**

Медицинский центр Бугандо, который является одной из четырех учебных многопрофильных больниц в Объединенной Республике Танзания, располагает отделением интенсивной терапии на 13 койко-мест, куда поступают все пациенты со столбняком. Официальные программы иммунизации против столбняка, действующие на территории Объединенной Республики Танзания, нацелены на детей или женщин.

**Осуществленные перемены:**

На основе информации, полученной из наших стационарных журналов, мы выявили шесть пациентов старше младенческого возраста со столбняком среди пациентов мужского пола с обрезанием в недавнем анамнезе. Только один из этих пациентов был обрезан в рамках субнациональной программы добровольного мужского медицинского обрезания. Остальные пять были обрезаны вне программы, например в небольших сельских диспансерах или традиционными специалистами по обрезанию крайней плоти без официального медицинского образования. Шесть пациентов были в возрасте 11–55 лет, из них пять (83%) умерли в больнице — все по причине развития генерализованного сепсиса.

**Выводы:**

Проведение обучения по обработке и гигиене раны в рамках танзанийской программы добровольного медицинского обрезания крайней плоти у мужчин, вероятно, способствует снижению заболеваемости столбняком в возрасте старше младенческого, связанным с проведением обрезания. Соответствующая заболеваемость среди мальчиков и мужчин, прошедших обрезание вне этой субнациональной программы, вероятно, выше. Рекомендуется обучать всех специалистов по обрезанию крайней плоти у мужчин надлежащей обработке и лечению ран, а также соблюдать программы вакцинации для обеспечения того, чтобы мужчины Танзании в раннем детстве проходили иммунизацию от столбняка.

## Introduction

By 2012, 14 countries in eastern and southern Africa – including the United Republic of Tanzania – had been prioritized for the scale-up of voluntary medical male circumcision.^1^ The goal of the scale-up was to reduce the transmission of human immunodeficiency virus (HIV) by circumcising over 35 million males in the priority countries by 2020.^1^ Only 13 cases of tetanus – one of which was Tanzanian, were attributed to the voluntary medical male circumcisions, that were performed in the 14 priority countries between 2012 and 2016.^2^ Since 2009, the United Republic of Tanzania has had a subnational programme of voluntary medical male circumcision that covers the Lake Zone in the north of the country. Between 2009 and 2015, this programme, which has received support from the United States Agency for International Development, the United States President’s Emergency Plan for AIDS Relief and other international donors, provided 497 259 circumcisions.^3^ Based on our clinical experience working in the intensive care unit at a Tanzanian referral hospital, we believed that non-neonatal male circumcision-related tetanus was being underreported in the United Republic of Tanzania. We therefore decided to conduct a detailed investigation of the patients with non-neonatal tetanus admitted to our hospital between 2001 and 2016, to see how many, if any, were related to recent circumcision.

## Local setting

Our study was conducted in the United Republic of Tanzania. We investigated tetanus patients admitted to the Bugando Medical Centre, which is one of just four referral teaching hospitals in the country. The Bugando Medical Centre is located in the city of Mwanza and serves 15 million people, who live in the Lake Zone. Throughout our study period, the Centre had a 13-bed intensive care unit that included seven ventilators.

The United Republic of Tanzania has been covered by the Expanded Programme on Immunization since 1975.^4^ Although the programme’s general coverage of its target Tanzanian population was estimated to be about 50% in the early 1980s,^4^ tetanus vaccine coverage among Tanzanian children younger than one year was estimated to have risen to 87.8% by 2010.^5^ The Tanzanian programme of tetanus vaccination is targeted at children younger than one year and women of childbearing age, i.e. women aged 14–49 years. In 2008, according to the Tanzanian Ministry of Health and Social Welfare, 80% of pregnant women received at least one dose of tetanus toxoid and 56% of women received at least two doses.^6^ There is no system in place to ensure that Tanzanian males receive any tetanus toxoid after their infancy, despite international recommendations for repeat tetanus vaccination every 10 years.^7^

## Approach

Patients admitted to Bugando Medical Centre are recorded prospectively in log books, together with their diagnoses and dates of admission. We investigated all patients admitted with a diagnosis of non-neonatal tetanus to Bugando Medical Centre’s intensive care unit between 1 May 2001 and 31 July 2016. Tetanus was diagnosed on clinical findings of rigidity, spasms and or trismus. All patients were managed in accordance with a standardized hospital protocol. Medical records were only available for patients admitted after 2008. For these patients, we recorded potential risk factors, e.g. age, report and timing of prior injury and or the presence and characteristics of a wound. We also determined if each male patient had been circumcised and, if so, when, where and by whom the circumcision had been performed.

We summarized categorical variables using percentages and compared their values using χ^2^ or Fisher’s exact tests, as appropriate. For continuous variables, we determined medians and interquartile ranges (IQR) and compared values with Wilcoxon rank-sum tests.

Ethical approval was obtained from the Bugando Medical Centre, the Tanzanian National Institute for Medical Research and Weill Cornell Medicine, New York, United States of America.

## Relevant changes

We identified 280 patients with non-neonatal tetanus who were admitted to Bugando Medical Centre’s intensive care unit during our study period. These patients, of whom 241 (86.1%) were male and 141 (50.4%) died while admitted, had a median age of 30 years (IQR: 19–46). The median age of the 139 survivors was significantly lower than that of the 141 fatal cases of tetanus (27.0 vs 36.5 years; *P* < 0.001).

We located medical notes for 162 of the 197 patients with non-neonatal tetanus admitted after 2008. Of the patients for whom medical notes were available, 159 (98.1%) had a recorded wound anywhere, 51 (31.4%) had a recorded wound on a leg, 46 (28.4%) a recorded wound on a foot and 22 (13.6%) had a recorded wound on a hand. Six (3.7%) of these patients were males who had a recent history of circumcision without, apparently, any other recent wound (Table 1). Five of these patients had circumcisions performed outside of the local subnational programme of voluntary medical male circumcision, i.e. at small rural dispensaries (2 men), in a traditional ceremony where the circumcision had been performed by traditional provider with no formal medical training (1 man) or in another, unspecified, location (2 men). The six patients identified as non-neonatal cases of circumcision-related tetanus were aged 11–55 years (mean: 29.8 years). Their first symptoms had been noticed one to two weeks after their circumcisions and they spent 1–22 days (mean: 8.8 days) in the intensive care unit before they died of overwhelming sepsis (5 men) or was discharged (1 man). Five of the six patients, all of whom died in the hospital, required invasive mechanical ventilation. Although the level of mortality among the patients with circumcision-related tetanus (5/6; 83.3%) was higher than that among the other patients with non-neonatal tetanus admitted after 2008 (99/191; 51.8%), the difference was not statistically significant (*P* = 0.09).

**Table 1:** Patients with associated with male circumcision after infancy, Bugando Medical Centre, Mwanza, United Republic of Tanzania, 2009–2016

Age, years	Time from circumcision to symptom onset, weeks	Year**^a^**	Time in ICU, days	Outcome	Home area	Place of circumcision	Wound condition
30	1	2009	22	Survived	Lamadi	Traditional ceremony^b^	Clean
21	1	2010	1	Died	Nyamoagolo	Unknown location^b^	Clean
11	2	2014	7	Died	Magu	Local dispensary^b^	Clean
18	1	2014	17	Died	Misungwi	Local dispensary^b^	Clean
44	2	2014	1	Died	Sengerema	Unknown location^b^	Clean
55	1	2015	5	Died	Sengerema	VMMC programme	Discharging pus

ICU: intensive care unit; VMMC: voluntary medical male circumcision.

^a^ No patients were recorded between 1 January 2016 and the end of the study period, on 31 July 2016.

^b^ Outside of VMMC programme.

Although the patient who developed tetanus after being circumcised within the programme of voluntary medical male circumcision was known to be HIV-infected, we could not find any information indicating whether he was using antiretroviral therapy. No information about other potential risk factors for post-circumcision tetanus – e.g. the application of cow dung to the wound^8^, was available.

## Lessons learnt

At one of the four Tanzanian referral hospitals, we detected six patients with circumcision-related non-neonatal tetanus over a seven-year period. Only one of those six patients had been circumcised within the local subnational programme of voluntary medical male circumcision and, in consequence, formally reported as an adverse event within the programme.^2^ Good wound care is essential for the prevention of infection post-circumcision.^9^ It seems likely that the incidence of tetanus within any formal programme of voluntary medical male circumcision is generally low because teaching on safe wound care accompanies most, if not all, such programmes. Our data highlight the need for a national policy that ensures that smaller dispensaries and other circumcision providers receive similar teaching on wound care. By inviting traditional providers to participate in training programmes, their knowledge, skills and willingness to engage with the more formal health services could be improved.^10^^,^^11^ The development and use of a formal protocol for the management of wound care, which has been shown to reduce wound infections in remote African settings,^12^ may also be beneficial in reducing tetanus infection after circumcision.

We support recent calls to integrate tetanus vaccination with adult male circumcision, as a way of boosting immune status in men.^13^^–^^15^ Formalization of tetanus vaccination programmes and establishment of tetanus as a notifiable disease have been associated with global declines in the incidence of tetanus since the 1940s.^14^ In the United States, for example, implementation of these two strategies appears to have led to a reduction in the annual incidence of tetanus from 500–600 patients in the 1940s to 25 in 2014.^14^ In the United Republic of Tanzania, where tetanus reporting is not mandatory, only two patients with non-neonatal tetanus were reported in 2014.^14^ The results of our research and another study based at the Bugando Medical Centre^13^ indicate that such tetanus is being underreported. In studies conducted since 2000, only 28% (40/145) of Tanzanian males aged at least 15 years were found to be seroprotected against tetanus^15^ and only 24% (24/102) of 102 tetanus patients at the Bugando Medical Centre reported previous immunization against the disease.^13^ Implementation of a robust immunization programme against tetanus, ideally coupled with mandatory tetanus reporting, could be very beneficial in the United Republic of Tanzania and should be a priority.

Our study had several limitations. First, we had no access to the medical records of patients admitted before 2009. Second, the patients we investigated are unlikely to be nationally representative because Bugando Medical Centre is only accessible to those who are able to pay for transport and treatment and well enough to travel to reach the hospital. It therefore seems likely that we missed many patients with tetanus that occurred, during our study period, within the catchment area of Bugando Medical Centre. Future prospective studies that include both regional and district hospitals are urgently needed.

In conclusion, we believe that the teaching of wound hygiene after circumcision and administration of tetanus vaccine at the time of adult circumcision have the potential to prevent both morbidity and mortality in young Tanzanian men (Box 1).

Box 1 **Summary of main lessons learnt**

- Within the Tanzanian programme of voluntary medical male circumcision, education on wound hygiene probably helps to reduce the incidence of non-neonatal circumcision-related tetanus.
- The corresponding incidence of circumcision-related tetanus among the boys and men who are circumcised beyond this subnational programme is probably higher.
- The training of all circumcision providers in wound care and a vaccination programme to ensure that male Tanzanians receive tetanus immunization after infancy would probably be very beneficial.
